# Case for diagnosis. Stewart-Treves syndrome after mastectomy^☆^

**DOI:** 10.1016/j.abd.2021.12.003

**Published:** 2022-07-08

**Authors:** Roberta Akeme de Oliveira Sato, Clovis Antônio Lopes Pinto, Celia Antonia Xavier de Moraes Alves, Juliana Arêas de Souza Lima Beltrame Ferreira

**Affiliations:** Department of Dermatology, Faculdade de Medicina de Jundiaí, Jundiaí, SP, Brazil

## Case report

A 56-year-old female patient reported the appearance of a nodule on the posterior region of the right arm six months before. During this period, she reported that the lesion showed progressive growth, pain, and friability, with frequent local bleeding. This patient had been submitted to a radical mastectomy and right axillary dissection, with adjuvant chemotherapy and radiotherapy 11 years before, and since then, she had chronic lymphedema in the right upper limb.

**Figure 1:**
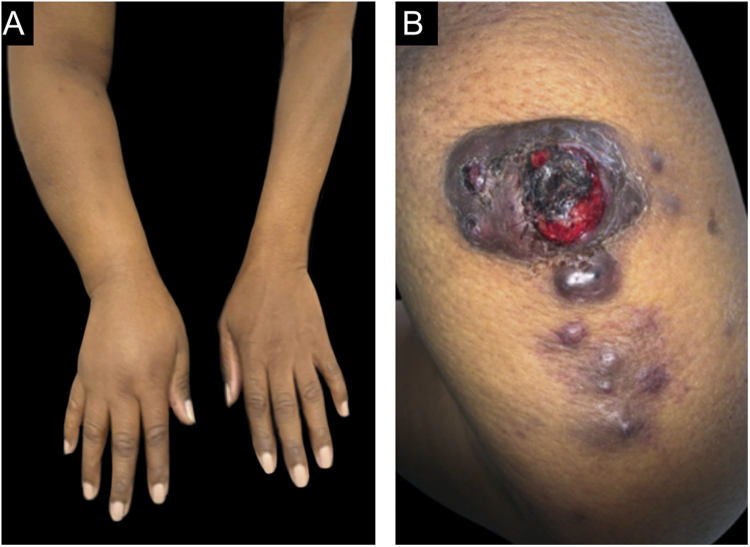
(A), Lymphedema in the right upper limb. (B), Tumor with ulcerated areas in the posterior region of the right arm, accompanied by satellite nodules.

Dermatological examination revealed a hyperchromic tumor, measuring approximately 5.0 × 4.0 cm, with central areas of ulceration and slight local bleeding on the posterior region of the right arm. Lymphedema was also observed in the right upper limb and violaceous-brown satellite nodules, measuring up to 0.5 cm (Fig. 1).

**Figure 2:**
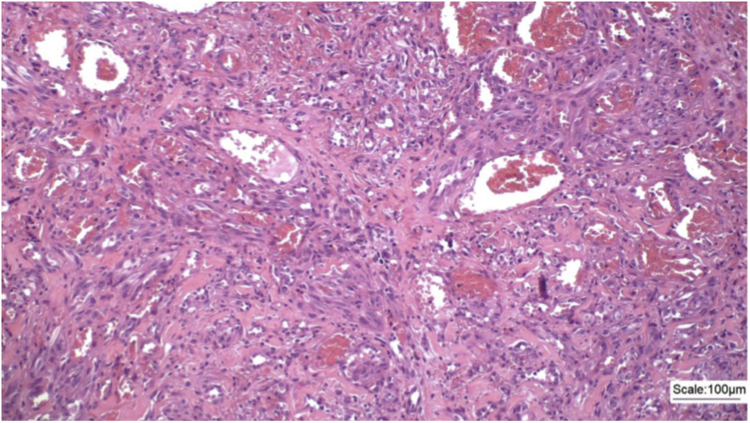
Proliferation of epithelioid and spindle cells, permeating malformed vascular channels and spaces, (Hematoxylin & eosin, ×100).

An incisional biopsy of the tumor and satellite nodules was performed. The patient returned for consultation after one month with a histopathological result that showed a neoplasm characterized by vascular formations permeated by atypical epithelioid cells infiltrating the superficial and deep dermis, with perineural permeation (Fig. 2).

**Figure 3:**
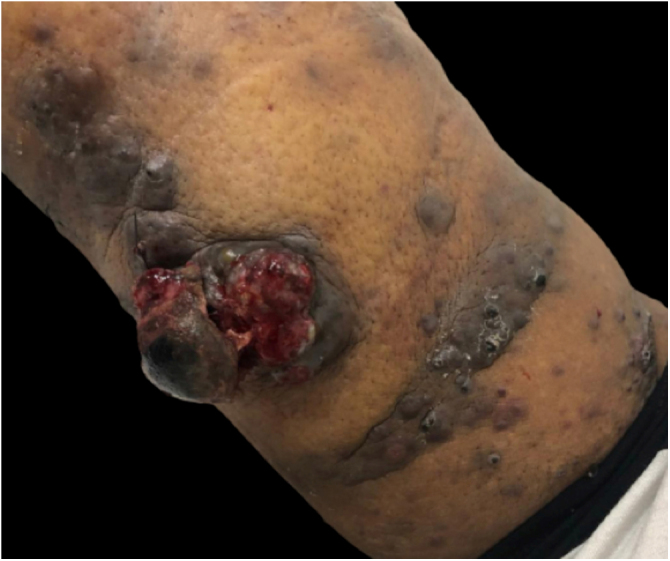
Significant increase in the number of satellite lesions and in the ulcerated area of the central tumor.

At this consultation the tumor was slightly larger and there was an increase in the number of satellite lesions (Fig. 3).

## What is your diagnosis?

a) Stewart-Treves syndrome
b) Squamous cell carcinoma
c) Melanoma
d) Merkel carcinoma

**Figure 4:**
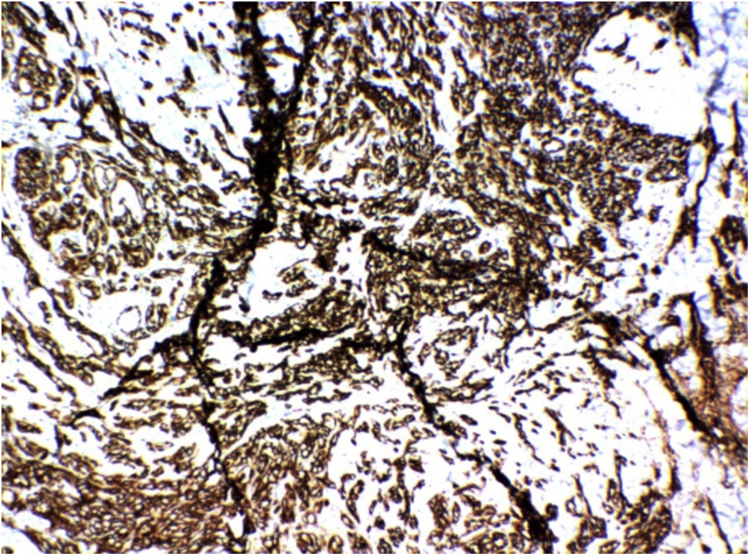
Immunohistochemistry showing positivity for CD31.

Immunohistochemistry examination was requested, which showed CD31 positivity, confirming the hypothesis of Stewart-Treves syndrome (Fig. 4).

## Discussion

Stewart-Treves syndrome (STS) is a rare entity, with a poor prognosis, consisting of the appearance of cutaneous angiosarcoma in areas of chronic lymphedema and accounts for approximately 5% of angiosarcomas.^1^, ^2^, ^3^, ^4^ Its occurrence is more commonly observed after a radical mastectomy with axillary dissection, with a latency period that varies from five to 11 years.^2^, ^4^ However, it can also appear in areas of chronic lymphedema due to venous stasis, morbid obesity, post-surgical procedures, lymphatic malformations, and chronic infections, among others.^2^, ^5^

The etiology and pathophysiology of the syndrome remain unknown. It is debated whether the lymphedema would favor oncogenesis due to the lymphatic drainage failure and interstitial fluid accumulation and stasis, in addition to the possible occurrence of neoplastic transformation during angiogenesis.^4^ They initially present as macules and papules, developing into nodules and tumors that can reach large volumes.^6^, ^7^ Satellite lesions, pain, and local bleeding are common.^6^

The diagnosis is based on the clinical picture, along with the biopsy. Histopathology may be suggestive, with irregular vascular spaces lined by mitotic hyperchromatic pleomorphic tumor endothelial cells.^4^ Immunohistochemistry helps in the diagnostic confirmation, with both CD31 and CD34 markers being positive.^4^

The treatment is based on surgical resection with wide margins, and chemotherapy and radiotherapy can also be used.^2^, ^3^, ^6^, ^7^ In the present case, the oncology service chose to use chemotherapy with docetaxel, and the oncology surgery team, so far has not indicated surgical treatment. The occurrence of distant metastases is not uncommon,^3^ with the lungs being the most commonly affected organs; the patient in the present case was submitted to CT scans of the chest, abdomen, and pelvis, which did not disclose the presence of metastases.

Although it is rare, knowledge of STS is necessary because, due to its aggressiveness, only its early diagnosis can help to increase patient survival.

## Financial support

None declared.

## Authors' contributions

Roberta Akeme de Oliveira Sato: Design and planning of the study; drafting and editing of the manuscript; critical review of the literature.

Clovis Antônio Lopes Pinto: Intellectual participation in the propaedeutic and/or therapeutic conduct of the studied cases; effective participation in research orientation.

Celia Antonia Xavier de Moraes Alves: Effective participation in research orientation; intellectual participation in the propaedeutic and/or therapeutic conduct of the studied cases.

Juliana Arêas de Souza Lima Beltrame Ferreira: Effective participation in research orientation; intellectual participation in the propaedeutic and/or therapeutic conduct of the studied cases; critical review of the manuscript; approval of the final version of the manuscript.

## Conflicts of interest

None declared.
